# Seed Mucilage Polysaccharides: A Comprehensive Review of Molecular Architecture, Green Extraction Technologies, Bioactivities, and Industrial Applications

**DOI:** 10.3390/foods15152616

**Published:** 2026-07-26

**Authors:** Afifa Aziz, Hadia Sehar, Muhammad Zeeshan Adil, Waseem Khalid, Kit-Leong Cheong

**Affiliations:** 1 Guangdong Provincial Key Laboratory of Aquatic Product Processing and Safety, Guangdong Ocean University, Zhanjiang 524088, China; afifaaziz44@gmail.com; 2 Guangdong Province Engineering Laboratory for Marine Biological Products, Guangdong Ocean University, Zhanjiang 524088, China; 3 Guangdong Provincial Engineering Technology Research Center of Seafood, Guangdong Ocean University, Zhanjiang 524088, China; 4 Guangdong Provincial Engineering Technology Research Center of Prefabricated Seafood Processing and Quality Control, Guangdong Ocean University, Zhanjiang 524088, China; 5 College of Food Science and Technology, Guangdong Ocean University, Zhanjiang 524088, China; 6 School of Food Science and Engineering, South China University of Technology, Guangzhou 510641, China; hadiasehar3@gmail.com (H.S.); mrzeeshan0300@gmail.com (M.Z.A.); 7 Department of Inorganic, Organic and Biochemistry, Faculty of Chemical Sciences and Technologies, University of Castilla La Mancha, 13071 Ciudad Real, Spain

**Keywords:** seed mucilage, bioactive compounds, extraction, food applications, polysaccharides

## Abstract

Seed mucilage is a natural hydrophilic polysaccharide gel secreted by seed coat epidermal cells comprising xylan, pectin, glucomannan, and cellulose alongside minor proteins, minerals, and lipids, with its composition varying substantially across plant species. This review critically compares conventional and green extraction technologies, including ultrasound-, microwave-, enzyme-, and subcritical water-assisted extraction, on a seed-mass-normalized yield basis together with their solvent-to-seed ratios and processing times, showing that green technologies can reduce solvent consumption, energy use, and processing time relative to matched conventional controls, though the magnitude of these gains is method and species-specific rather than uniform. We synthesize how molecular weight, uronic acid content, branching pattern, and microfibril organization govern seed mucilage hydration, rheological, emulsifying, and foaming behavior, and link these structure–function relationships to its antioxidant, prebiotic, anti-inflammatory, and antimicrobial bioactivities. Beyond functional characterization, this review evaluates the translational barriers, extraction standardization, safety and regulatory status, and clinical validation that currently limit seed mucilage adoption in food, pharmaceutical, and cosmetic industries. Unlike previous reviews focused on ecological function or application cataloging, this review uniquely reframes seed mucilage as a structurally programmable bioactive polysaccharide, integrating molecular architecture, green process intensification, and structure–function–application relationships to bridge fundamental chemistry with industrial translation.

## 1. Introduction

The rising demand for bio-based materials that are sustainable, biodegradable, and functional has driven research to focus on underutilized plant-based biopolymers. A natural hydrophilic polysaccharide gel exuded by the seed coat epidermal cells, called seed mucilage, has been identified as a promising natural polysaccharide candidate for use, owing to its structural complexity, physicochemical versatility, and bioactive properties. Plant-based mucilage is a thick, gelatinous substance primarily composed of complex polysaccharides. It is produced by nearly all plants and certain microorganisms (e.g., bacteria, fungi) [1,2]. Mucilage is a natural hydrophilic biopolymer, meaning it readily attracts and binds water, which allows it to perform vital functions in plants such as water storage, seed germination, and wound healing [3,4]. Mucilage can be found in various plant parts including seeds, roots, leaves, and bark [5]. Seed mucilage is secreted from the seed’s coat outer epidermis and is particularly rich in hydrophilic polysaccharides [6]. The composition of seed mucilage, composed of pectin, cellulose, and hemicellulose, varies with species and genotype, affecting mucilage functional properties [2,7]. The mucilage content of seeds typically includes polysaccharides (78.4%), proteins (7.3%), minerals (5.6%), and lipids (3.1%) [8]. Upon hydration, mucilage expands and forms a gel-like capsule around the seed [9,10]. This expansion can dramatically increase the size of the seed. For example, *Alyssum minus* seeds have mucilage that is three times greater, and the mass of the mucilage increases 167 times after inhibition [11]. This characteristic is also observed in species from families such as *Plantaginaceae*, *Lamiaceae*, and *Brassicaceae* [12]. Ecologically, seed mucilage also plays a vital role in seed germination, hydration, dormancy regulation, and interaction with the surrounding soil matrix, as well as helping to fix the seed to the soil, preventing it from being washed away [2,7]. Seed mucilage also helps in seed adhesion to substrates (myxospermy), distribution of seed by animals, water, or microbial colonization, influencing the plant–soil–microbe interaction. The development of seed mucilage is seen as a special trait that enhances the plant’s adaptation to diverse environmental conditions, specifically arid and semi-arid ecosystems [12]. In the present studies, conventional and advanced extraction and analytical techniques were used to determine which techniques enhance the yield, efficiency, and preserve bioactive properties.

In history and tradition, seed mucilage has been used in numerous food, agricultural and medicinal applications. Plant seeds containing mucilage have a long history of use in traditional medicine, valued for healing properties and used in traditional and casual practices [13]. For over 4000 years, traditional Persian medicine has used seed mucilage for therapeutic properties to alleviate various ailments. These applications, connected to ancient knowledge and their scientific valuation, expose the uses in modern medicine [14]. For instance, mucilages from seeds such as *Linum usitatissimum* (flaxseed), *Plantago ovata* (psyllium), and *Salvia hispanica* (chia) have long been utilized as stabilizers in food and herbal formulations; their mucilaginous properties are also used in treating gastrointestinal disorders, promoting skin hydration, and serving as natural thickeners [15,16]. These traditional applications laid the foundation for the contemporary interest in seed mucilage as a multifunctional biomaterial for food, pharmaceutical, and cosmetic industries. Several reviews have previously addressed specific facets of seed mucilage. Tsai et al. [17] provided a comprehensive synthesis of its ecological and biological functions, seed dispersal, germination, and abiotic stress tolerance, together with an outlook on genetic engineering approaches, but did not address extraction technology, structural characterization, or industrial translation. More recently, Gupta et al. [1] surveyed the functional properties and application landscape of mucilage from several seed species, including film formation, wound-dressing, and drug-delivery uses. However, neither review provides a quantitative, technique-level comparison of green extraction methods on a standardized basis, nor systematically links molecular architecture, monosaccharide composition, branching pattern, and molecular weight, to functional and bioactive outcomes across seed species. This gap has practical consequences: without such a framework, extraction and purification process choices remain empirical rather than structure-informed, slowing industrial translation. The present review addresses this gap by (i) critically comparing conventional and green extraction technologies, including ultrasound-, microwave-, enzyme-, and subcritical water-assisted extraction, on a seed-mass-normalized yield basis, together with their processing intensities (solvent-to-seed ratios, treatment times, energy demand); (ii) synthesizing structure–function–application relationships across seed species rather than presenting them as isolated case studies; and (iii) evaluating the translational and regulatory barriers, safety, purity standards, and scale-up economics that determine whether laboratory findings reach food, pharmaceutical, and cosmetic markets. In this sense, the review is oriented toward researchers and process engineers developing green extraction and functional-ingredient applications, rather than serving as a further descriptive catalog of seed mucilage sources.

This review therefore examines the structural architecture of seed mucilage, current and emerging preparation techniques, including subcritical water, enzyme-, microwave-, and ultrasound-assisted extraction, its physicochemical and bioactive properties, and its applications across the food, pharmaceutical, and cosmetic sectors. By critically synthesizing recent literature and identifying unresolved research gaps, this review aims to bridge fundamental mucilage chemistry with its industrial translation. Relevant literature was identified through systematic searches of Scopus, Web of Science, PubMed, and Google Scholar, for studies published between 2015 and 2025 using combinations of the keywords “seed mucilage,” “plant mucilage,” “green extraction,” “ultrasound-assisted extraction,” “microwave-assisted extraction,” “subcritical water extraction,” “enzyme-assisted extraction,” “mucilage bioactivity,” and “mucilage rheology.” Studies were included if they reported original data on seed mucilage composition, extraction, characterization, functional properties, bioactivity, or application; non-English-language articles, conference abstracts without peer review, and studies concerning non-seed mucilage sources (e.g., cactus pad, fruit, or bacterial exopolysaccharide mucilage) were excluded unless cited for direct structural or functional comparison.

## 2. Botanical Origin and Biological Function of Seed Mucilage

Seed mucilage is produced by seeds of diverse plant families, with particularly rich representation in *Brassicaceae*, *Lamiaceae*, *Plantaginaceae*, and *Linaceae* [12,18,19]. This trait, in which seeds produce a hydrophilic mucilage layer upon hydration, is termed myxospermy and is documented in over 80 angiosperm families [12]. Ecologically, seed mucilage plays vital roles in plant survival and reproduction [2,7]. Upon hydration, mucilage expands dramatically; for example, *Alyssum minus* seeds show a 167-fold increase in mucilage mass after imbibition [11]. This expansion fixes seeds to the soil matrix, preventing them from being washed away and promoting germination in arid and semi-arid ecosystems. The model plant *Arabidopsis thaliana* (*Brassicaceae*) exhibits intricate molecular mechanisms for the synthesis and secretion of seed mucilage. Its mucilage is dominated by pectin, specifically rhamnogalacturonan-I (RG-I), which accounts for approximately 90% of the polysaccharide content, together with minor hemicellulose (xylan, galactoglucomannan) and cellulose fractions [12]. Mucilage in *Arabidopsis* is composed of various cell wall polysaccharides, including pectin, hemicellulose, and cellulose. The primary pectin domain in *Arabidopsis* mucilage is rhamnogalacturonan [20]. Structurally, Arabidopsis mucilage separates into two distinct layers on hydration: an outer, non-adherent layer that readily dissolves in water, and an inner, adherent layer that remains attached to the seed coat and is uniquely reinforced by cellulose microfibrils [21]. This organization contrasts with species such as basil (*Ocimum basilicum*), whose mucilage is dominated by galactoglucomannan rather than pectin, and chia (*Salvia hispanica*) and flax (*Linum usitatissimum*), whose mucilages are xylan/heteroxylan-rich with a substantial pectin fraction, illustrating that the pectin-dominant Arabidopsis model does not generalize across families and that extraction and functional behavior must be interpreted species by species rather than assumed from the model system. *Arabidopsis* contains at least 54 genes that have been identified as crucial for the synthesis, secretion, and modification of this mucilage [22]. For instance, the CELLULOSE SYNTHASE 5 (*cesa5*) gene plays a crucial role in the proper formation of mucilage layers, as *cesa5* mutants result in altered mucilage morphology [21]. Furthermore, members of the Class II KNOX transcription factor family, namely KNAT3, and KNAT7, are involved in regulating mucilage production during the primary developmental stages of *Arabidopsis* seeds [23].

**Figure 1:**
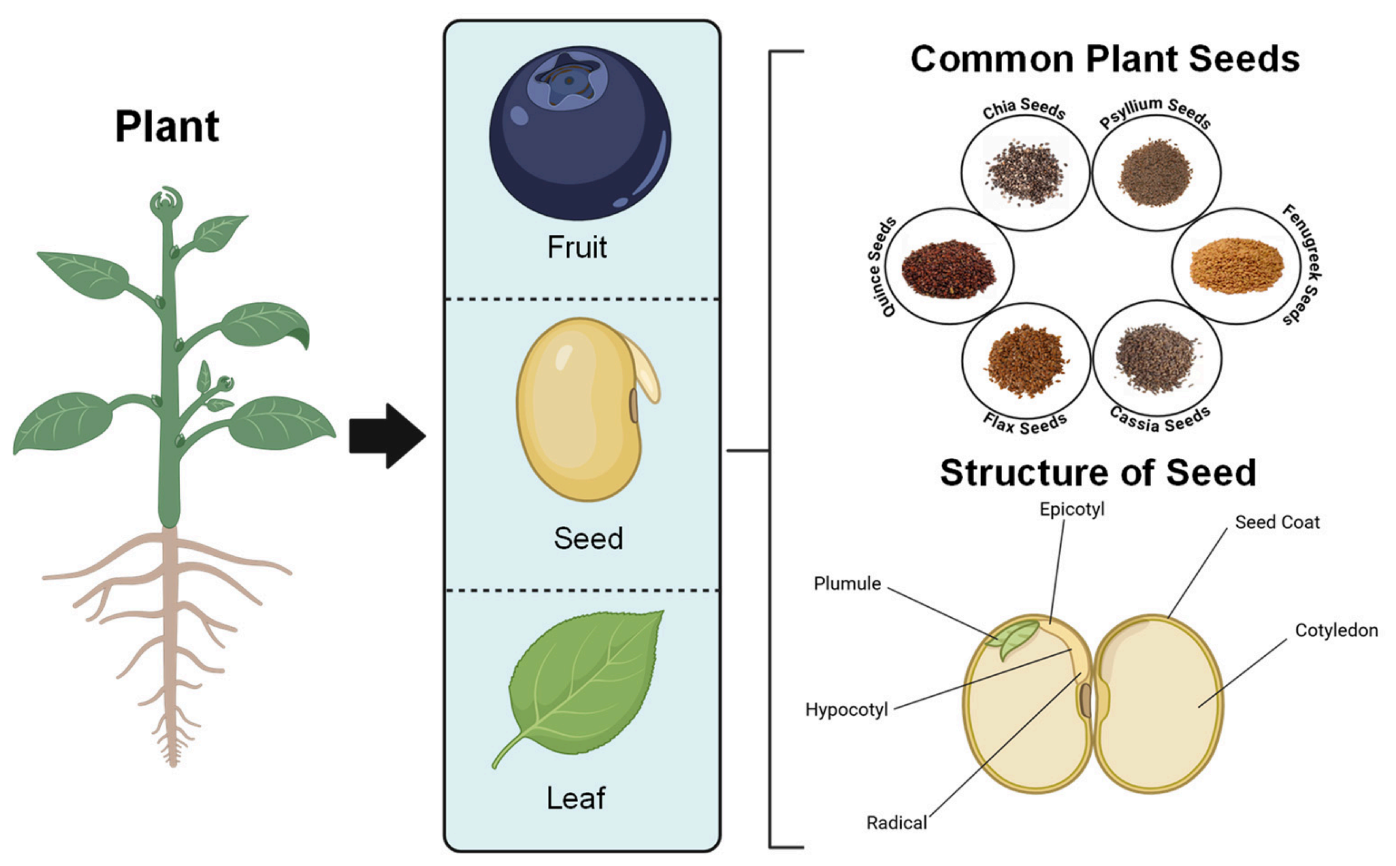
Brief representation of the plant anatomical sources of biopolymers, with an emphasis on the seed structure relevant to mucilage isolation.

Psyllium (*Plantago ovata*) seeds are one of the most commercially unique sources of mucilage. The seed husk of psyllium, which consists primarily of mucilage polysaccharides, is widely used in laxative and dietary fiber supplements [24]. This mucilage forms a polysaccharide-rich gel around the seeds upon hydration, a phenomenon known as myxospermy [25]. The dry husk layer that undergoes mucilaginous transformation, commonly called psyllium or isabgol, is industrially separated and milled from the seeds of *Plantago ovata* [26]. Its composition and functional properties are distinct from those of the *Brassicaceae* [27]. Flax (*Linum usitatissimum* L.), a member of the *Linaceae* family, has seeds serving as a significant source of mucilage famous for its versatile uses [28]. The *Lamiaceae* family, commonly known as the mint family, comprises a diverse group of plant species, including basil (*Ocimum basilicum* L.) and chia (*Salvia hispanica* L.), In recent years, these species have gained scientific and industrial interest due to the valuable functional properties of their seeds, particularly their mucilage content [29,30,31,32]. The sources and the distribution of mucilage in the different parts of the plants are illustrated in Figure 1. These mucilages have complex polysaccharides located in the outermost layer of the seed coat and possess rheological properties that make them distinct by-products of the flax oil and fiber industries [18,19]. Collectively, this botanical diversity is not incidental—it is the reason extraction yield, molecular architecture, and downstream functional performance vary so substantially across seed species, a relationship examined in detail in Section 3 and Section 4 [32,33].

## 3. Molecular Architecture and Structural Characterization

### 3.1. Chemical Composition and Primary Structure

Seed mucilage is a polysaccharide-dominated biomaterial, with polysaccharides typically accounting for approximately 78.4% of the total composition, followed by proteins (7.3%), minerals (5.6%), and lipids (3.1%) [8]. The polysaccharide fraction is compositionally diverse, comprising cellulose, glucans, xylans, mannans, galacturonans, and rhamnogalacturonans, with the relative proportions of each varying considerably among species [34]. Enzymatic proteins embedded within the mucilage matrix contribute to post-secretory modification of these polysaccharides [35]. In Arabidopsis thaliana, for example, pectins, principally rhamnogalacturonan-I and homogalacturonan, comprise approximately 90% of mucilage content, with the remaining 10% consisting of cellulose, structural proteins, and hemicelluloses such as galactoglucomannan and xylan [36]. Upon hydration, this polysaccharide network forms a hydrophilic gel capsule around the seed [2,17], a structure that supports seed dispersal, soil adhesion, and protection against abiotic stress during germination [37].

Mucilage composition is not uniform across species or even genotypes within a species [9]. Upon hydration, seed mucilage typically forms a two-layered structure comprising an outer non-adherent layer and an inner adherent layer [37]. This layered architecture is observable in species like *Arabidopsis thaliana* [38]. Structurally, seed mucilage represents a modified derivative of the seed coat cell wall, built from cellulose, pectins, and hemicelluloses that collectively determine its viscosity, adhesion, and water-retention properties [12,39,40]. In addition to these dominant polysaccharides, proteins and lipids are also present in smaller but functionally relevant proportions [2]. Pectins, particularly rhamnogalacturonan I (RG-I) and homogalacturonan (HG), are typically the dominant components [41]. In Arabidopsis, both the water-soluble outer layer and the adherent inner layer are predominantly RG-I [12]. RG-I can assemble into highly branched, long-chain configurations that self-organize into diverse higher-order structures [42]. Homogalacturonan, the most abundant pectin type, is modified by pectin methylesterases (PMEs), which regulate its methylesterification level and thereby influence mucilage extrusion [43]. Hemicelluloses, principally xylans and glucomannans, together with cellulose and structural proteins, further shape mucilage architecture [20,39]. While cellulose itself forms a microfibril network specifically localized to the inner adherent layer in Arabidopsis [21,44]. Other monosaccharide components identified in various seed mucilages include D-glucose, D-galactose, D-mannose, L-arabinose, D-xylose, and L-rhamnose, alongside uronic acids such as D-galacturonic and D-mannuronic acids [8].

#### 3.1.1. Monosaccharide Composition and Glycosidic Linkages

**Figure 2:**
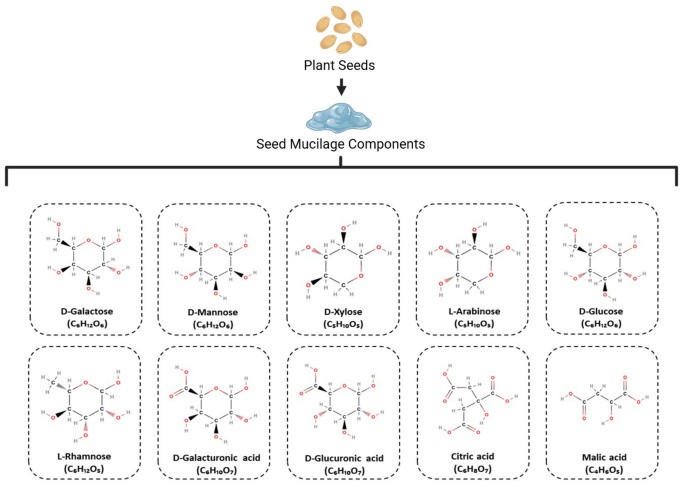
Chemical constituents and monomeric building blocks of seed mucilage.

Complex polysaccharides present in mucilage are composed of monosaccharides, which cannot be further broken down, such as pentose sugars and methylpentose sugars that are linked to organic acids, such as uronic acid, by glycosidic linkages [45]. Generally, nine different monosaccharides such as D-glucose, D-galactose, D-mannose, L-arabinose, D-xylose, and L-rhamnose, alongside uronic acids such as D-galacturonic and D-mannuronic acids, can be identified in seed mucilage using different detection approaches as described in Table 1 [8,46]. The most common mucilage polysaccharides, such as pectin and hemicellulose, are composed of building blocks of two polymers called rhamnogalacturonan and arabinoxylans, respectively [47]. RG-I consists of alternating α-(1,2)-rhamnose and α-D-(1,4)-galacturonic acid disaccharide units, while arabinoxylans are built from repeating β-1,4-linked xylose units [48], as described in Figure 2. In basil seed mucilage, the specific combination of mannose, glucose, and galactose monomers, uronic acid content, and glycosidic linkage pattern is largely responsible for its gel-like consistency [49], whereas *Plantago ovata* mucilage is dominated by xylan/arabinoxylan with arabinose side chains [50]. Because monosaccharide identity and linkage pattern together determine gelation and viscosity behavior, this compositional layer is a necessary starting point for interpreting the functional properties discussed in Section 6.

#### 3.1.2. Associated Molecules: Uronic Acids, Proteins, Phenolics, and Molecular Weight

Quantitative uronic acid assays confirm the pectic character of many seed mucilages, with galacturonic acid content correlating with overall structural complexity. The polyelectrolyte behavior and gelling capacity of the heteroxylans in *Plantago ovata* husk, for instance, derive from the charged acidic residues of galacturonic acid [50,59]; consistent with this, FTIR analysis of basil seed mucilage identified uronic acid signatures via asymmetric and symmetric C-OO^−^ stretching at 1600 and 1400 cm^−1^, respectively [60]. Proteins, sometimes regarded as impurities rather than functional constituents, nonetheless contribute to the texture and consistency of mucilage-containing food products [61], and their content varies substantially across species: from 1.04% in mesquite (*Prosopis* spp.) to 20.9% in quince (*Cydonia oblonga*), with intermediate levels reported in basil (2.01%) [62], Qodume Shirazi (*Alyssum homolocarpum*, 17.9%) [63], Qodume Shahri (*Lepidium perfoliatum*, 4.6%), chia (*Salvia hispanica*, 2.08%) [56,64], cress (*Lepidium sativum*, 2.54%) [65], balangu (*Lallemantia royleana*, 2.71%) [55], and *Plantago major* (6.66%) [66]. This range underscores the pronounced biochemical diversity among mucilage-producing species. FTIR is the most commonly used method for detecting this protein fraction, for example, the 1456 cm^−1^ absorbance band in basil seed mucilage spectra is attributed to protein content [67].

Phenolic compounds, a diverse class of plant secondary metabolites, are also present in seeds and their mucilage [68], with their composition and concentration depending on growing conditions, extraction method, species, and genotype [69]. Beyond their antioxidant contribution, phenolic compounds also influence polymeric cross-linking and emulsifying behavior in mucilage [68]. Molecular weight distributions are typically resolved by high-performance size-exclusion chromatography (HPSEC) or gel permeation chromatography (GPC). Kaczmarczyk et al. (2023), for example, used GPC to resolve two arabinoxylan populations in *Plantago ovata* extracts, at 200 and 1780 kDa [50]. Cassia uniflora mucilage, measured by light-scattering intensity, showed a molecular weight of 514 ± 110 kDa, intermediate between acacia seyal gum (206–928 kDa) and gum tragacanth (840 kDa) [70], illustrating that molecular weight varies over more than an order of magnitude even among structurally related plant gums. Extraction conditions are a major driver of this variability, since mechanical, thermal, and enzymatic treatments can fragment or crosslink polysaccharide chains differently; this relationship, and its consequences for industrial performance, is examined quantitatively in Section 4.

### 3.2. High-Order Structure and Morphology

**Figure 3:**
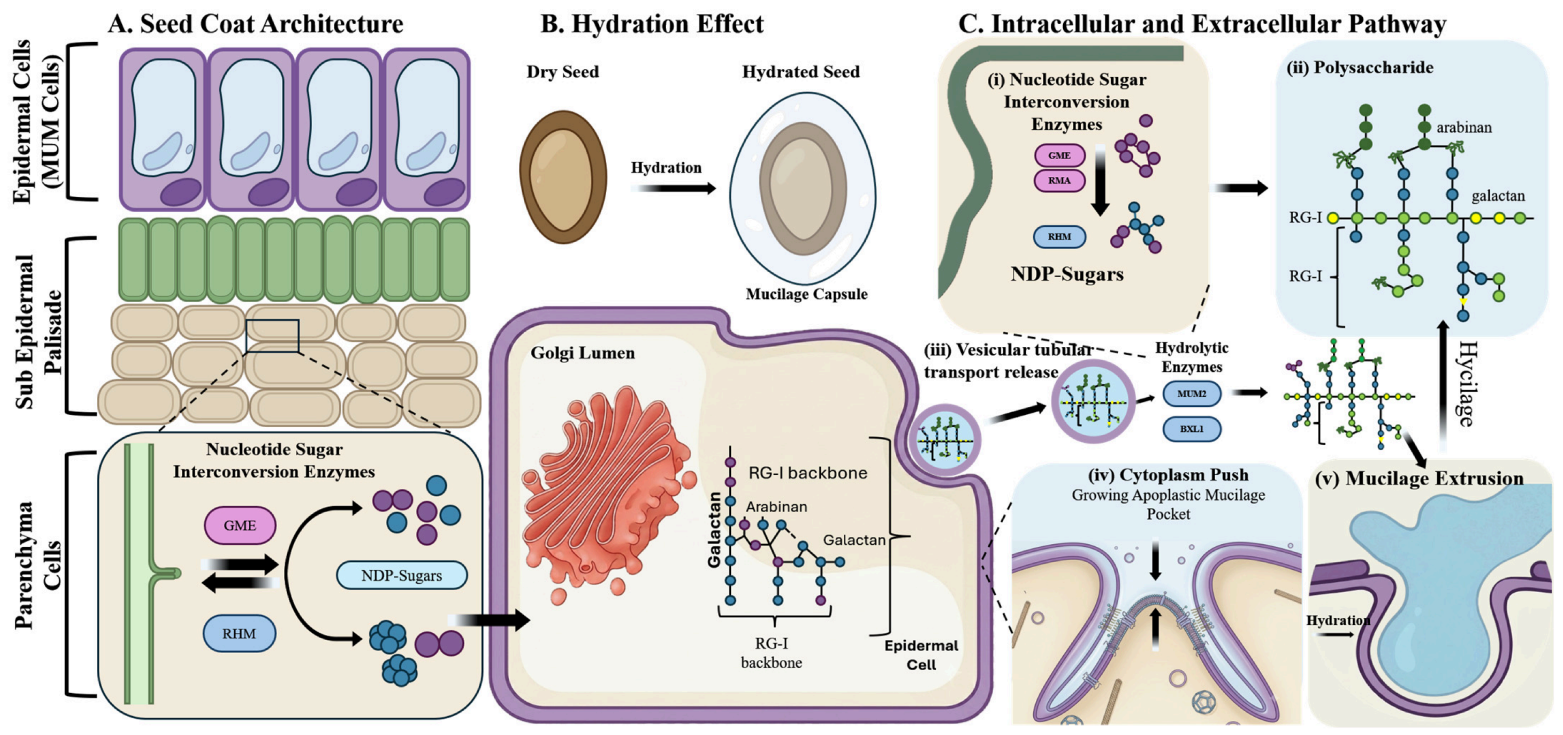
Schematic representation of seed mucilage biosynthesis and extrusion. Molecular and cellular pathway of mucilage biosynthesis in seed coat epidermal cells. (**A**) Seed coat architecture showing the epidermal cell layer containing mucilage-producing cells, with subepidermal palisade and parenchyma layers beneath. (**B**) Upon hydration, mucilage is secreted from the seed coat, forming a non-transparent gel-like capsule. (**C**) Intracellular and extracellular biosynthesis pathway: (i) Nucleotide sugar interconversion enzymes in the Golgi membrane synthesize NDP-sugars; (ii) complex polysaccharides (RG-I backbone with arabinan/galactan side chains) assemble in the Golgi lumen; (iii) the polysaccharide complex is transported to the apoplast where hydrolytic enzymes (MUM2, BXL1) modify side chains; (iv) the apoplastic mucilage pocket expands, pushing the cytoplasm into a volcano-like structure; (v) upon seed hydration, mucilage is extruded to the seed surface.

Upon hydration, seed mucilage typically separates into a two-layered structure: an outer, non-adherent, water-soluble layer and an inner, adherent layer reinforced by cellulose microfibrils [21,22,38]. Biosynthesis, summarized in Figure 3, begins in the Golgi apparatus, where nucleotide sugar interconversion enzymes generate activated sugar donors (NDP-sugars) that are assembled into RG-I backbones (galacturonic acid and rhamnose) with arabinan or xylan side chains via specific glycosyltransferases [71]. Pectic polymers are synthesized in the Golgi while cellulose is synthesized at the plasma membrane; both are exported and deposited into an apoplastic mucilage pocket formed by polar secretion from seed coat epidermal cells [72]. Upon hydration, pectin swelling generates internal pressure that ruptures the outer radial cell wall and extrudes the gel-like mucilage. In some species, temporal layering occurs during secretion, RG-I is deposited first on the upper face, followed by arabinoxylan and glucan in deeper layers, establishing an internal architecture that influences later release mechanics. This process is under genetic control at each stage (synthesis, secretion, post-secretory modification): at least 54 genes have been implicated in Arabidopsis mucilage biosynthesis [22], including MUM-family genes governing wall modification required for extrusion, and CELLULOSE SYNTHASE 5 (CESA5), which is specifically required for correct inner-layer formation [21]. Collectively, this tightly coordinated biosynthetic sequence links monosaccharide-level composition to the functional roles of mucilage in water retention and germination.

#### Advanced Analytical Techniques for Structure Elucidation

**Figure 4:**
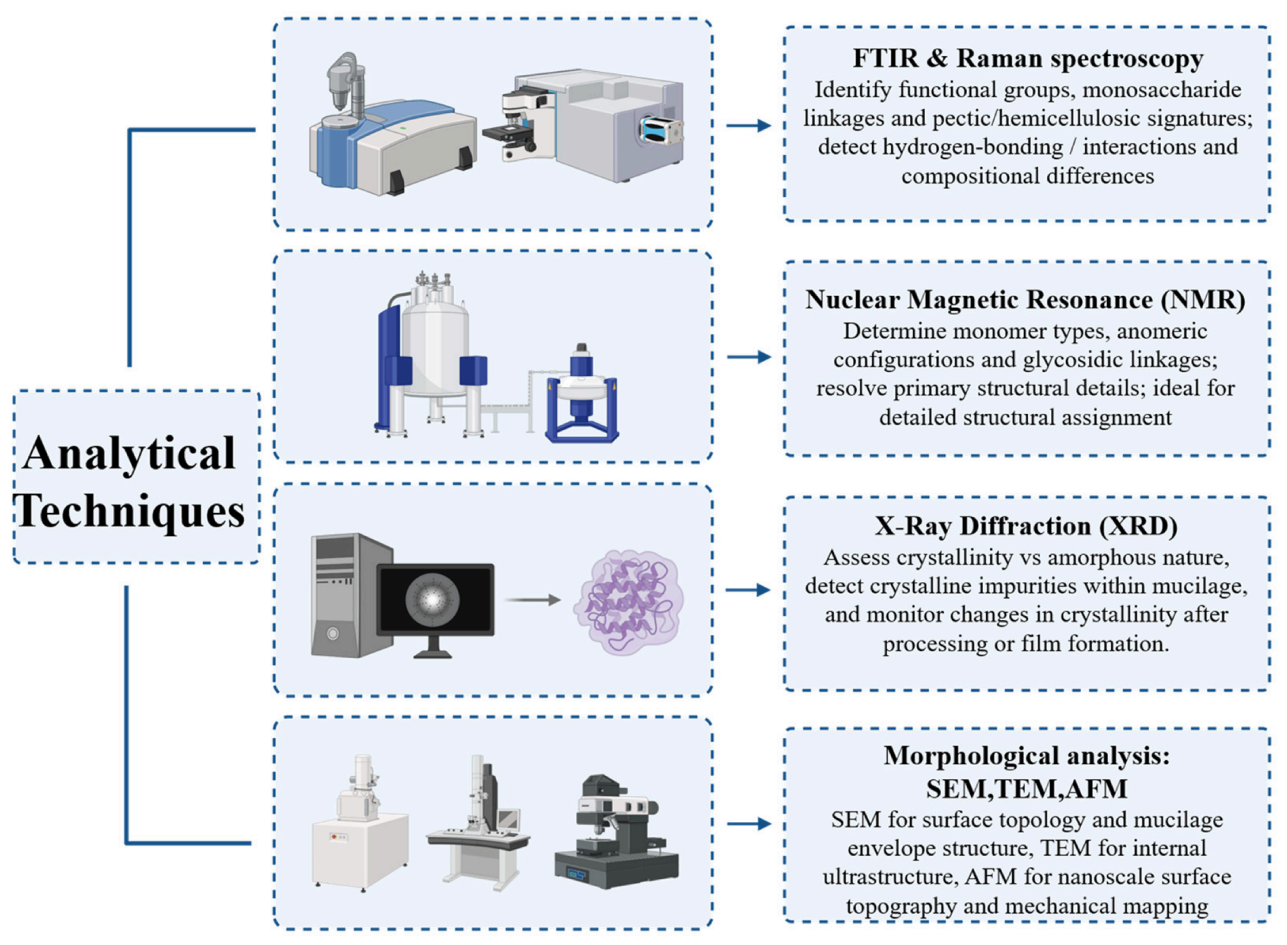
Multi-technique workflow for seed mucilage characterization. FTIR and Raman spectroscopy identify functional groups and glycosidic linkages; NMR resolves monomer types, anomeric configurations, and linkage positions; XRD assesses crystallinity and structural changes; and SEM, TEM, and AFM provide hierarchical morphological analysis from surface topology to nanoscale mechanical mapping.

Comprehensive structural elucidation of seed mucilage requires spectroscopic, diffraction, chromatographic, and thermal characterization methods used in combination, since no single technique resolves molecular composition, conformation, and morphology simultaneously (Figure 4).

The Fourier transform infrared (FTIR) spectroscopy and Raman spectroscopy are considered vital for fast, deep, and information-based characterization of mucilage. FTIR analysis is used for mapping out cellular components such as lipids, proteins, and carbohydrates [73,74]. FTIR analysis of yellow pitahaya mucilage, for example, resolved hydroxyl-group absorption (3250 cm^−1^), C–H bonds in CH_2_ groups (3120 and 2988 cm^−1^), amide bonds from protein (1739 cm^−1^, low intensity), and the carboxylic signature of galacturonic-type uronic acids (1600 cm^−1^) [75,76,77,78]. Raman spectroscopy, which measures frequency shifts in scattered light upon interaction with sample molecules [79], showed comparable spectral patterns (strong signals at 2800–3000 cm^−1^) for Opuntia ficus-indica and Aloe vera mucilage, consistent with their shared polysaccharide-rich composition [80]. Because both techniques probe vibrational/functional-group signatures, they are largely complementary in application (FTIR for polar functional groups, Raman for symmetric/non-polar bonds) rather than providing independent structural confirmation [81].

Nuclear Magnetic Resonance (NMR) is non-destructive and resolves anomeric configuration, linkage position, and ring type at the atomic level [82], information that neither FTIR nor Raman can provide. NMR analysis confirmed rhamnose, xylose, arabinose, mannose, and fructose in Manilkara zapota mucilage [83], and fully resolved the linkage sequence of chia seed polysaccharide, a backbone of mannose, glucuronic acid, and xylose connected via β-D-Manp-(1→,→6)-α-D-GlcpA-(1→,→2,4)-β-D-Xylp-(1→,→6)-β-D-Manp-(1→linkages, dominated by (1→6) and (1→2,4) connections [84].

Morphological characterization, such as Atomic Force Microscopy (AFM), Transmission Electron Microscopy (TEM) and Scanning Electron Microscopy (SEM), links structural composition to functional performance in ways spectroscopic methods cannot, since none of the above techniques resolve three-dimensional architecture. SEM imaging of chia seed mucilage revealed a porous, fibrous network with entangled polymeric strands and heterogeneous pore sizes, directly explaining its water-holding capacity and gel-forming behavior [85]; similar long, tangled, parallel cellulose fibril arrangements have been observed across species [9,41,86]. TEM, though applied less frequently, resolves nanoscale fibril dimensions below SEM’s resolution limit, for example, revealing a multidirectional fibril network with ~20 nm diameter in *Salvia hispanica* mucilage [87]. AFM, which avoids the destructive sample preparation SEM/TEM require, additionally captures nano-mechanical behavior: AFM analysis of quince seed mucilage showed that ultrasonic treatment reduced both fibril diameter and length, directly linking a specific processing variable to nanostructural change [58].

Taken together, these six techniques are complementary rather than redundant: FTIR and Raman establish chemical functional-group identity; NMR resolves linkage-level molecular structure; XRD distinguishes crystalline from amorphous character; and SEM, TEM, and AFM provide morphology across decreasing length scales (micro- to nanoscale), with AFM uniquely preserving native hydration state. No single technique provides a complete structural picture, which is the principal current limitation of the field, and most published characterizations rely on two or three methods rather than the full suite, limiting cross-study comparability (Table 2).

## 4. Extraction Process Intensification: From Seed to Polysaccharide

**Figure 5:**
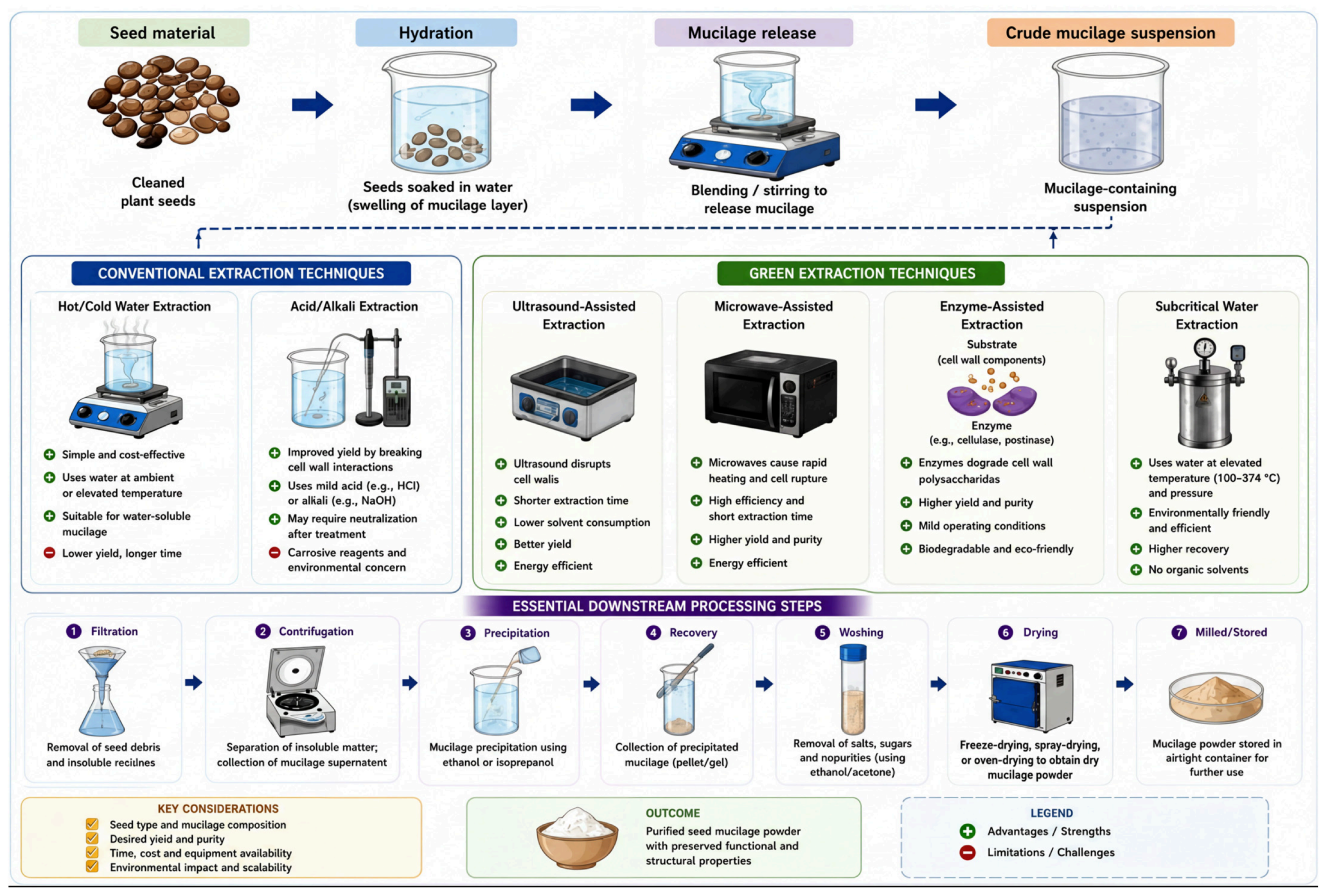
Comparative overview of conventional and extraction techniques for isolating mucilage from plant seeds, alongside essential downstream processing steps.

In recent years, a paradigm shift from conventional to green extraction technologies has been driven by the need for more sustainable, efficient, and product-preserving processes. Extraction is a critical determinant not only of mucilage yield but also of the structural integrity, purity, and techno-functional properties of the final polysaccharide product. Seed mucilage extraction methods are broadly categorized into conventional and green technologies, as illustrated in Figure 5. A note on yield comparability, applicable throughout this section: mucilage “yield” is reported in the literature on at least three distinct bases: (i) mass of extracted mucilage relative to initial seed mass, (ii) mass of extracted polysaccharide relative to the mucilage or gum already present in a pre-processed material (e.g., seed husk powder), and (iii) mass of purified/precipitated solid relative to the crude liquid extract. These are not interchangeable, and a high percentage under basis (ii) or (iii) does not indicate superior performance over a lower percentage under basis (i). Wherever possible, the basis is stated explicitly below; where a source does not report it, this is flagged rather than assumed. Acid and alkali treatments are valuable conventional tools for modifying the yield, structure, and composition of extracted mucilage, but they are not classified as green extraction technologies. True green technologies, such as ultrasound-, microwave-, and enzyme-assisted extraction, and subcritical water extraction, are defined by their ability to improve efficiency while reducing solvent use, energy consumption, and processing time relative to conventional methods [92,93,94].

### 4.1. Conventional Extraction and Purification

Conventional techniques are the traditional processing methods long used in food science and biotechnology to evaluate the nutritional, sensory, and physicochemical properties of food matrices prior to the adoption of advanced techniques [92]. For seed mucilage, these are primarily water- and acid/alkali-based, typically combined with heat, physical agitation, and precipitation to isolate the polysaccharide [5]. Water extraction is broadly used across plant, algal, fungal, and microbial matrices to recover bioactive compounds, proteins, phenolics, and polysaccharides. Temperature is a key optimization variable [95], and defines the two principal sub-methods. Hot-water extraction (HWE) uses temperatures of 70–100 °C, or reflux/autoclaving conditions up to 121 °C; 80 °C has been reported as optimal for Plantago major and flaxseed [92,96], while makatan and moringa seeds require higher temperatures (100 °C) [97]. Elevated temperature enhances solubility and disrupts cell wall structure, promoting release of intracellular material into the solvent [92]. Cold-water extraction (CWE) instead immerses seeds in water at 4–25 °C, relying on diffusion and solubilization of water-soluble compounds while minimizing thermal degradation; this typically yields a product with reduced viscosity and surface tension. The light brown/cream color typical of conventionally extracted mucilage reflects deactivation of polyphenol oxidase during the heating step [98].

Acid/alkaline-assisted extraction (AAE) enhances yield and modifies the functional properties of extracted hydrocolloids by chemically breaking down cell wall components [99,100]. Extraction efficiency depends on seed type, temperature, pH, and duration [101]. For example, Plantago major mucilage extraction is optimized at pH 6.8 [96], while Trigonella elliptica mucilage extraction is optimized at pH 6.90, 86.1 °C, and an 8.3 g seed weight [102]. AAE typically requires high temperature and long processing time to be effective [103]. Despite their simplicity, conventional methods are time- and energy-intensive and often yield incompletely, since mucilage is not fully detached from the seed surface. Mechanical shearing can also introduce impurities from damaged seed particles, requiring additional purification that further increases processing cost, which is a limitation that directly motivates the shift to green technologies below.

### 4.2. Emerging Green Extraction Technologies

To overcome the limitations of conventional methods, a range of “green” or emerging technologies have been developed. These techniques leverage physical or enzymatic processes to enhance extraction efficiency, minimize degradation of the target polysaccharide, and reduce environmental impact. Green extraction techniques are rapidly evolving, sustainable, eco-friendly methods that increase yield and preserve the functional properties of valuable biopolymers [93,94]. These technologies aim to enhance the recovery of bioactive compounds from natural sources like plants, algae, and microorganisms, which decreases the environmental impact, energy consumption, and solvent use. Green extraction is connected with the principles of green chemistry and sustainability, emphasizing safety, efficiency, and waste reduction [104,105].

#### 4.2.1. Ultrasound-Assisted Extraction

Ultrasound-assisted extraction (UAE) uses high-frequency sound waves to induce cavitation in the solvent, generating localized high pressure and shear forces that disrupt cell structure and enhance mass transfer [106,107]. Because it can operate with water alone and at moderate energy input, UAE is generally considered a clean, green technology [108]. A critical distinction, often collapsed in the literature, is equipment type: probe-type (horn) sonication delivers substantially higher acoustic power density than bath-type sonication, and yields reported under one system are not directly comparable to the other. Chia seed mucilage extracted by combined heat/probe-sonication at 50–80 °C for 30–60 min (seed-to-water ratio 1:30) achieved 6.92–10.52% yield (by seed mass), versus 1.03–1.86% for heat alone, which is a three- to five-fold improvement attributable specifically to probe-type cavitation [109]. This exemplifies a broader pattern in green extraction: process intensification that increases yield or speed frequently does so by fragmenting the polysaccharide backbone, with direct consequences for downstream rheological performance (Section 6). Camelina seed UAE has also been combined with hydrodynamic forces in a tubular reactor/recirculating pump configuration [110]. Optimized UAE of Plantago major gum (70 °C, 40 min, 90% ultrasonic power) achieved a maximum yield of 13.1%, while UAE of Citrus grandis seed increased yield by 59% relative to conventional buffer extraction [109]. As with chia, UAE efficiency depends critically on power, frequency, time, and temperature, which must be optimized jointly to avoid excessive degradation of high-molecular-weight chains [111].

#### 4.2.2. Microwave-Assisted Extraction

Microwave-assisted extraction (MAE) selectively heats polar molecules within the plant matrix and solvent, producing rapid internal temperature rise that disrupts cell structure and promotes bioactive-compound release [112]. Several studies have optimized MAE parameters for efficient recovery of mucilage from various seed sources. For instance, in the case of *Ocimum basilicum* var. *album* seeds, MAE yielded a maximum extraction efficiency of 17.00 ± 0.14% under optimized conditions of 4.33 min extraction time, 760 W microwave power, and a solvent-to-sample ratio of 20:1 mL/g [113]. MAE has also shown a significant increase in extraction yield and enhanced the emulsifying properties of Camelina sativa seed mucilage [114]. MAE offers several key advantages, including remarkably short extraction times, high selectivity, and improved product quality [93,94]. Data from studies on basil seed mucilage (*Ocimum basilicum* var. *album* L.) demonstrate the superior performance of MAE. Using optimized conditions of 4.33 min, 570.32 W power, and a 40:1 solvent-to-sample ratio, MAE achieved a yield of 17.01%, which was significantly higher than the yield from a conventional extraction method carried out at 50 °C for 1 h [114]. Moreover, SEM analysis revealed that the MAE-extracted mucilage had a more porous structure, and FTIR confirmed that the structural integrity of its functional groups was preserved [114]. However, MAE carries two notable caveats: elevated temperatures can drive Maillard browning, producing a dark product color undesirable for colorless-ingredient applications, and intensive microwave exposure can alter primary structure, shift spectroscopic signatures, and reduce particle size in ways that affect functional performance [93,94].

#### 4.2.3. Enzyme-Assisted Extraction

Enzyme-assisted extraction (EAE) uses cellulases, xylanases, glucanases, pectinases, and proteases to selectively degrade cell wall components, improving mucilage accessibility under mild processing conditions [115,116]. This method leverages specific enzymes to selectively degrade cell wall components, which enhances the release of polysaccharides, including mucilage from the seed matrix [117]. Enzymes such as cellulases, xylanases, glucanases, pectinases, and proteases play a vital role in breaking down these complex carbohydrate structures and proteins, making the mucilage more accessible for extraction [118,119]. The application of xylanase and glucanase, either individually or in combination, significantly increased the yield of mucilage from *Asplenium australasicum*, increasing it from 3.64% in control groups to 6.04–6.86% under EAE [115]. This method is highly selective and operates under mild conditions, preserving the integrity of the extracted polysaccharide. This method’s high selectivity and mild operating conditions preserve polysaccharide integrity, but enzyme choice is critical: EAE using the cellulase preparation Celluclast generally reduced molecular weight and intrinsic viscosity across extraction procedures, though this specific preparation caused the least degradation among those tested—making it comparatively more suitable where high-purity, high-molecular-weight mucilage is required [120].

#### 4.2.4. Subcritical Water Extraction

Subcritical water extraction (SWE) uses water held liquid under elevated pressure at 100–374 °C, exploiting the resulting changes in dielectric constant and viscosity to act as a tunable solvent for both polar and non-polar compounds [121,122,123,124]. SWE offers high diffusion rates and mass transfer relative to other methods, generally translating into shorter processing times [122]. It has been applied to oil extraction from cottonseed, palm kernel, and *Butia odorata* seeds [125,126]. For tamarind kernel powder, SWE at 175 °C achieved a 62.28% recovery yield [127]. This expressed relative to the mucilage/gum content already present in the tamarind kernel powder feedstock, not relative to seed mass; it is therefore not directly comparable to the seed-mass-normalized yields reported for UAE, MAE, and EAE above, and should not be read as evidence that SWE is inherently more efficient at extracting mucilage from whole seed. Independent of this comparability issue, SWE’s high operating temperature has real functional costs: reduced viscosity, pasting properties, molecular weight, and water absorption index in the extracted xyloglucan, alongside enhanced water solubility, consistent with thermal chain degradation, and a darker product color from Maillard reactions and caramelization, disadvantageous where a light-colored ingredient is needed [127]. SWE has also been applied to rambutan seed polysaccharides, where defatting pretreatment measurably improved yield [128]. Its principal advantages remain low operating cost, environmental sustainability, and elimination of toxic organic solvents [129].

Green extraction technologies are defined by their ability to improve efficiency and reduce environmental impact by reducing solvent use, energy consumption, and processing time [111]. Overall, no single green technology dominates across all performance criteria (Table 3): UAE and EAE best preserve molecular weight and functional integrity at moderate yield gains; MAE offers the fastest processing but risks thermal/Maillard degradation; and SWE achieves the highest recovery efficiency from pre-processed feedstocks at the cost of chain degradation and color formation. Selecting among them therefore requires matching the technology to the intended application, such as high-purity pharmaceutical-grade mucilage that favors UAE/EAE, while bulk industrial recovery may tolerate SWE’s degradation trade-offs.

### 4.3. Downstream Processing: Purification and Drying

Purification removes impurities such as minerals, proteins, and other insoluble components, improving the functional properties of the isolated mucilage [49,137,138]. Filtration, precipitation, and centrifugation are the most common approaches [5,131,132]. For *Opuntia ficus-indica*, purification typically follows hot-water extraction with filtration to remove solid residues, then precipitation using organic solvents such as ethanol or isopropanol [139]. Precipitation solvent choice affects the yield of this purification step specifically, as isopropanol gave a 23.3% recovery versus 19% for ethanol [139], a yield measured relative to the crude aqueous extract, not to the original seed mass; it should not be conflated with extraction yield when comparing across the methods discussed above. Dialysis and ultrafiltration provide further purification and concentration options [133].

Drying is a critical downstream step affecting the physical, chemical, and functional properties of the final product [135,140], and method choice involves clear trade-offs. Freeze drying, which is sublimating ice from the frozen mucilage solution under vacuum [33], best preserves structural integrity, solubility, and emulsifying capacity by avoiding thermal exposure [97,134]. The spray drying technique converts the liquid feed into a dry powder by atomizing it into a hot drying medium [131,135]. It is a rapid and scalable method, but the high temperature can affect the mucilage properties [33]. Spray drying can effectively be used to encapsulate probiotic bacteria with chia and flaxseed mucilage, enhancing their viability and stability [141]. Oven drying is cost-effective but can reduce fiber content, solubility, and emulsion activity/stability at the high temperatures typically required [134]. Vacuum and air drying offer lower-cost alternatives with their own limitations, for example, vacuum drying is comparatively slow, and air drying is both slow and yields lower-quality product. Natural drying can significantly influence seed viability and moisture content over time. In *Syzygium cumini* seeds, prolonged drying was associated with a decline in both germination rate and moisture content [136].

Collectively, extraction, purification, and drying together determine the final quality of seed mucilage (Table 3), and each stage requires independent optimization: a high-yield extraction step can be undermined by a degradative drying step, and vice versa. Careful, application-specific selection across all three stages, not just extraction, is therefore necessary to preserve bioactivity and tailor mucilage for its intended end use [96,142].

## 5. Physicochemical and Functional Properties

**Figure 6:**
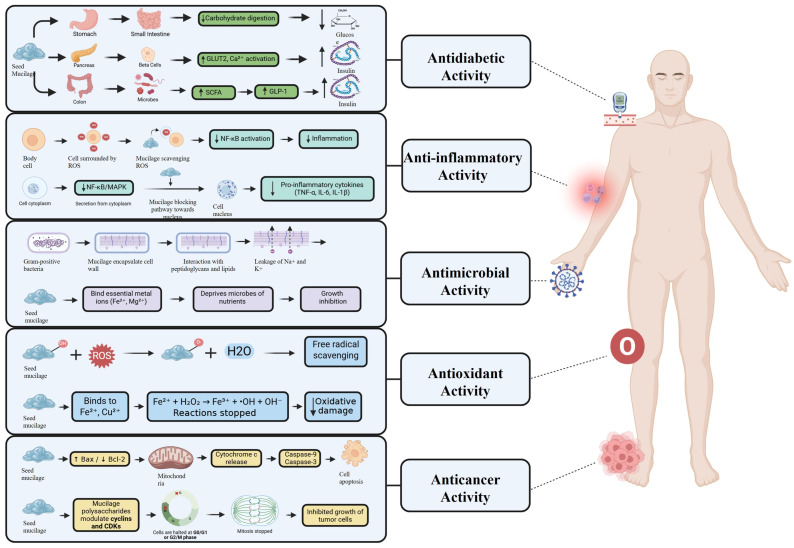
Mechanistic pathways illustrating the key bioactivities of seed mucilage within the human physiological system.

The molecular architecture described in Section 3, monosaccharide composition, branching pattern, uronic acid content, and molecular weight, directly determine the functional properties discussed below, summarized schematically in Figure 6.

### 5.1. Hydration Capacity

Hydration capacity, which is the mass of water retained by a sample under gravitational or applied external force, is a critical parameter governing the texture, stability, sensory character, and application performance of seed mucilage [143]. Reported values vary enormously across species: basil seed mucilage (97.5 ± 2.4 g water/g), *Cordia myxa* mucilage (14.94 ± 2.44 g water/g), tamarind seed mucilage (0.18 ± 0.014 g water/g), *Opuntia dillenii* mucilage (4.00 ± 0.10 g water/g), green gram mucilage (0.630 g water/g), karaya seed mucilage (0.670 g water/g), and crude durian seed mucilage (1.395–2.740 g water/g) [144,145,146,147,148]. This is a greater than 500-fold range, and it is not incidental: it tracks closely with the compositional diversity established in Section 3. Basil mucilage’s exceptionally high hydration capacity is consistent with its glucomannan-dominated, highly hydroxylated structure and large hydrodynamic volume, whereas tamarind’s low capacity reflects its more compact, xyloglucan-based architecture with fewer exposed hydrophilic side chains. Uronic acid content is a further driver: pectin-rich, RG-I-dominated mucilages carry a higher density of ionizable carboxyl groups, which increases both charge repulsion within the polymer network and its capacity to entrain water. Hydration capacity is also temperature-dependent within a given species; for tamarind seed mucilage, raising the extraction/measurement temperature from 25 °C to 65 °C increased water-holding capacity to 1.07 ± 0.025 g/g, confirming that processing conditions, not composition alone, modulate this property [143]. Owing to this tunable water-holding behavior, mucilage is valued as a natural swelling agent for improving water retention in fruit, vegetable, and dairy-based products, and is further exploited in nanofiber fabrication for filtration media, controlled-release delivery systems, and edible films [149].

### 5.2. Rheological Property

Rheological behavior is central to mucilage’s functional identity and provides indirect insight into its structural composition [150]. The high density of polar and charged functional groups in mucilage polysaccharides enables extensive water absorption, producing viscous, gel-like solutions [151]. Molecular weight is a primary determinant of this behavior, with viscosity and shear-thinning intensity scaling with chain length: reported molecular weights include quince seed mucilage (9610 kDa), flaxseed (1200 kDa), chia seed (800–2000 kDa), Arabic gum (250–1000 kDa), Mimosa pudica gum (2500 kDa), xanthan gum (2000–20,000 kDa), and basil seed (2320 kDa) [48,76,151,152,153,154,155,156]. Quince seed mucilage’s molecular weight is thus among the highest reported for a seed-derived hydrocolloid, comparable to or exceeding flaxseed, chia, Arabic gum, and basil, and falling within (rather than exceeding) the broad range reported for microbially derived xanthan gum. This exceptionally high molecular weight, combined with its pectin-rich, highly branched architecture, makes quince seed mucilage a particularly effective thickening, gelling, and binding agent in food formulations [157]. Beyond molecular weight, gel strength is governed by structural rearrangement and hydrogen-bonding extent within the polysaccharide network [158]. Gels are classified as strong (storage modulus G′ exceeding loss modulus G″) or weak (the reverse); weak gels flow more readily under applied stress, and most seed mucilages fall into this category [159,160]. Gel strength is further modulated by salt type, polymer concentration, temperature, and pH: flaxseed mucilage gel strength increases with higher dissolution temperature, increasing pH (6–9), and greater mucilage concentration, while different salt types exert variable, and not uniformly predictable, effects on gelation [159]. Quince seed mucilage shows particularly high gelling ability and hydrodynamic volume, reflecting its high molecular weight and branching density noted above, and is consequently valued as a stabilizing and gelling ingredient in food applications [161].

### 5.3. Emulsifying Properties

Seed mucilage contributes to emulsification by adsorbing at oil–water interfaces, modulating aqueous-phase viscosity, and sterically/electrostatically preventing droplet flocculation [162,163]. In quince seed mucilage, emulsification is attributed to carboxylic functional groups conferring a net negative surface charge and consequent electrostatic repulsion between droplets, producing emulsions with high reported stability (94.89%) [161,164]. Other seed mucilages show substantial variation in this property: cress seed mucilage (92%), Plantago seed mucilage (67.4%), flaxseed gum (86.84%), xanthan gum (89.28%), guar gum (93.90%), and Qodume Shahri (*Lepidium perfoliatum*) seed mucilage (93.17%) [96,144,165,166]. This functional versatility underlies the broad use of plant-derived polysaccharides, such as gums, mucilages, and carboxymethyl cellulose (CMC), as emulsifying agents across the food industry [167,168].

### 5.4. Foaming Property

Foaming ability, which is the capacity to generate and stabilize gas bubbles within a solution, depends on a mucilage’s surface activity, specifically its ability to form a cohesive film at the air–liquid interface [169,170]. This behavior is influenced by molecular weight, structural arrangement, chemical composition, and environmental conditions. Quince seed mucilage shows only average foaming ability but comparatively high foam stability relative to other mucilages [161]. Reported values illustrate the trade-off between these two parameters across species: flaxseed mucilage shows 75% foaming capacity, while Plantago seed mucilage shows 88.4% foaming stability [96,143]. Taken together, foaming and emulsifying behavior allow selection of species-specific mucilages matched to particular food-formulation requirements, for example, favoring higher-foam-stability sources for aerated products and higher-emulsion-stability sources for beverage or dressing applications.

## 6. Bioactive Properties and Health Promoting Pathways

Polysaccharide-rich mucilage matrices exhibit a broad spectrum of bioactive and functional properties, including antioxidant, prebiotic, anti-inflammatory, immunomodulatory, antimicrobial, antidiabetic, anticancer, cholesterol-lowering, and wound-healing/skin-protective activities [1,14,17], summarized mechanistically in Figure 6 and by seed source in Table 4. These activities derive directly from mucilage’s complex chemical composition, such as polysaccharides, uronic acids, proteins, lipids, and associated phenolic/flavonoid compounds, established in Section 3 [49,171].

### 6.1. Antioxidant Activities

Seed mucilage has one of the primary bioactive properties like antioxidant activity. Flaxseed mucilage is known for its antioxidant capabilities [132]. Similarly, *Ximenia americana* seed mucilage has demonstrated antioxidant activities linked to its phytochemical components [172]. Mucilage containing phenolic compounds and flavonoids is known for free radical scavengers contributes a significant antioxidant effect [49,182]. For instance, red rice has a flavonoid called proanthocyanidins, which is associated with strong antioxidant activity [182]. Moringa seed mucilage also contains glucosinolates and phenolic compounds, which contribute to its bioactive properties [183].

### 6.2. Prebiotic Properties

Seed mucilage also functions as a prebiotic substrate. Mucilages from chia, mustard, flaxseed, and fenugreek positively influence the gut microbial community, promoting the growth of beneficial taxa such as Bifidobacterium and Lactobacillus, genera with recognized importance for gut health across the life course [178]. This activity is consistent with the broader relationship between polysaccharide macromolecules, gut microbiota diversity, and short-chain fatty acid (SCFA) production, which together support metabolic and immune function; this relationship has been particularly well studied in the context of childhood obesity [184].

### 6.3. Anti-Inflammatory and Immunomodulatory Properties

Beyond antioxidant and prebiotic activity, seed mucilages exhibit measurable anti-inflammatory and immunomodulatory effects. *Moringa oleifera* mucilage, for example, shows immunomodulatory activity through inhibition of inflammatory mediators such as prostaglandins and cytokines, thereby reducing oxidative stress [185]. Moringa-derived bioactive polysaccharides further stimulate beneficial gut bacteria, including *Bifidobacterium*, *Phascolarctobacterium*, *Coprococcus*, and *Roseburia*. The resulting modulation of gut flora, and the associated increase in SCFA production, is considered critical for sustaining both immune function and gut barrier integrity [185,186].

### 6.4. Antimicrobial Properties

Food safety remains a major concern in food product development: foodborne illness affects an estimated 1 in 10 people globally each year (with incidence reaching up to 30% in some industrialized countries) [187,188]. Several food-derived proteins and polysaccharides possess intrinsic antimicrobial activity, including cress, chia, okra, and quince seed mucilages [164,189,190,191]. This property has been exploited in composite antimicrobial materials: antibacterial zinc oxide nanoparticles (ZnO-NPs) combined with xanthan gum, chitosan, or cellulose produce antagonistic action against harmful microbes while improving structural properties in various product formats [192,193,194]. When combined with basil seed mucilage specifically, ZnO-NPs have been used to develop wound dressings with enhanced antimicrobial activity, improved water retention, and higher solubility, which are the properties further enhanced when borax was incorporated as a cross-linking agent [195]. Collectively, these findings indicate that seed mucilage’s antimicrobial activity is readily exploitable across food, pharmaceutical, and biomedical materials applications.

## 7. Industrial Applications

**Figure 7:**
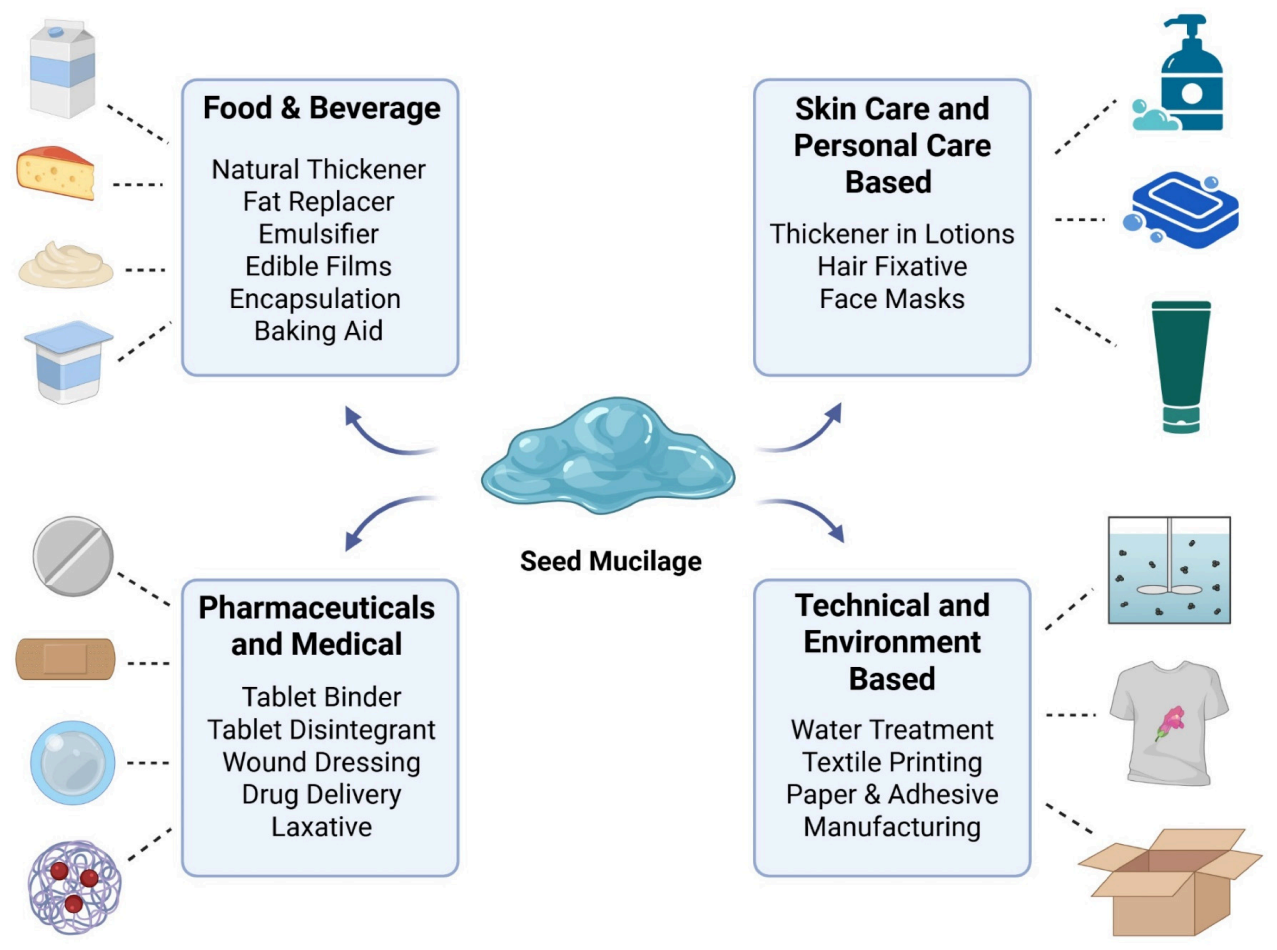
Applications of seed mucilage biopolymers across various industrial and technical sectors.

The growing demand for natural, eco-friendly ingredients has propelled seed mucilage’s development as a functional biopolymer across the food, pharmaceutical, cosmetic, and environmental sectors [173], illustrated in Figure 7. Its multifunctional profile, such as water-holding, emulsifying, film-forming, gelling, and mucoadhesive capacity, stems directly from the structural and rheological properties established in Section 3 and Section 5. In the food sector, seed mucilages function as stabilizers, thickeners, and edible coating/packaging materials, offering eco-friendly alternatives to synthetic additives [14,196]. *Lallemantia iberica* seed mucilage incorporated with *Malva sylvestris* leaf bioactive compounds has been used to produce bio-edible coatings that extend the oxidative stability of turkey meat [197], and chia seed mucilage coatings have improved shelf life and quality in eggs [198]. Beyond coatings, seed mucilage is increasingly explored as a base material for biodegradable edible films and packaging, which is a category directly relevant to the sustainability rationale of this review, and one that closely tracks the properties established in Section 5. Basil seed mucilage forms elastic, UV-absorbing, thermally stable biodegradable films suitable as edible packaging [199,200]; flaxseed mucilage films, built from its rhamnogalacturonan-I/arabinoxylan backbone (Section 3), show water vapor permeability and mechanical properties competitive with synthetic low-density polyethylene films [201,202]; and quince and chia seed mucilage films show tunable barrier and antioxidant properties as glycerol plasticizer concentration is varied, with chia films additionally incorporated with essential oils to confer antifungal activity for active packaging applications [203,204,205]. Collectively, these findings position mucilage-based films as a genuine alternative to petroleum-derived packaging, directly connecting this review’s extraction and structural chapters to a concrete sustainability application. Seed mucilages are also used to fabricate food-grade aerogels, serving as carrier matrices for functional ingredients owing to their high surface area and nanoporous structure [206,207], which protect sensitive compounds from adverse processing conditions and enhance stability [207]. Camelina seed mucilage, for example, has been used to fabricate bio-aerogels with a high surface area (240 m^2^/g) and nanoporous structure (6 nm) for food applications [206].

Seed mucilage’s high viscosity, swelling capacity, and mucoadhesive character (Section 5.1 and Section 5.2) make it a well-established natural excipient in solid and mucosal drug delivery systems. As tablet binders and disintegrants, fenugreek seed mucilage has demonstrated favorable disintegrant properties [208], and *Lepidium sativum* (cress) seed mucilage has shown effective binding at low concentrations, performing comparably to established binders such as HPMC and PVP K-30 [209,210]. Flaxseed mucilage has similarly been evaluated as a drug-release retardant polymer in matrix tablet formulations [211]. As a mucoadhesive polymer, fenugreek seed mucilage has been developed into a nasal drug delivery system for diazepam, with mucoadhesive strength, viscosity, and in vitro release performance exceeding conventional synthetic mucoadhesives such as HPMC and Carbopol 934 in the cited comparison [208], while *Plantago ovata* (psyllium) mucilage has been formulated into fast-disintegrating tablets [212]. This positions seed mucilage as a genuine, cost-effective alternative to synthetic pharmaceutical excipients, directly reinforcing the sustainability argument made for its food and packaging applications above, but for the pharmaceutical sector specifically. Seed mucilage and mucilage-derived plant extracts can be nanoencapsulated to enhance skin penetration and improve treatment efficacy for skin disorders, leveraging the antioxidant, anti-inflammatory, and antimicrobial activities established in Section 6 [213,214]. Beyond encapsulation, mucilage’s high water-holding capacity and film-forming behavior make it a natural candidate for moisturizing and thickening agents in topical cosmetic formulations, paralleling its established role as a wound-dressing component.

Mucilages from *Aloe vera* and *Opuntia ficus-indica* have been used for water purification, reducing turbidity through their coagulant/flocculant properties [215]. This environmental application, combined with the food-grade aerogel and biodegradable packaging applications above, illustrates that seed mucilage’s industrial value extends beyond direct product formulation into process-level and environmental remediation roles. Despite this broad application landscape, seed mucilage’s industrial and commercial uptake remains constrained by regulatory and economic barriers rarely addressed in the mucilage literature. With the notable exception of long-established sources such as psyllium husk, which carries FDA-recognized health claims for cholesterol and cardiovascular disease-risk reduction, most plant and seed mucilages currently lack approved food-additive status from either the U.S. Food and Drug Administration (FDA) or the European Food Safety Authority (EFSA), and the approval process required to obtain such status is both costly and administratively demanding [32,216,217]. This regulatory gap, rather than any inherent technical limitation, is a principal barrier to mucilage displacing established commercial hydrocolloids (e.g., xanthan gum, guar gum) at industrial scale, even where its functional performance is comparable or superior (Section 5). Compounding this, the extraction and purification cost analyses needed to assess commercial viability, addressed critically in Section 4, are rarely reported alongside functional-performance data in the primary literature, making direct cost–benefit comparison across mucilage sources difficult. Closing this regulatory and economic gap is likely to be the single largest determinant of whether seed mucilage moves from laboratory-scale characterization to genuine industrial substitution for synthetic or conventional hydrocolloid ingredients, a theme this review returns to in Section 8.

## 8. Current Challenges and Limitations in Seed Mucilage Research and Application

### 8.1. Ineffective Extraction and Purification

Traditional mucilage extraction methods, though widely used, remain inefficient, time-consuming, and waste-generating [217]. For instance, when psyllium seed husk was used directly for biofilm preparation without a multi-step extraction process, complete separation of mucilage from the seed proved difficult [218]. Extraction requirements are also species-specific: ultrasound-assisted extraction is considered near-essential for biofilm-grade mucilage preparation but does not generalize across all seed types (Section 4). Impurities such as minerals and alkaloids further complicate the process, requiring additional purification steps that add cost and processing time; basil seed mucilage extraction, for example, is complicated specifically by the need to remove these impurities during production [5]. As established in Section 4, this points to a genuine unresolved need for extraction protocols standardized enough to allow direct cross-study comparison, rather than the largely bespoke, species-specific optimization currently reported throughout the literature.

### 8.2. Flexible Structural Characteristics

Seed mucilage’s structural complexity, arising from its variable composition of cellulose, xylan, pectin, and glucomannan (Section 3), makes comprehensive structural characterization inherently difficult, since the relative proportions and functional consequences of these components vary substantially by species [40]. Current analytical techniques, while individually informative, lack the combined resolution needed to fully characterize this compositional and conformational diversity across seed sources [218]. For example, *Plantago ovata* seed mucilage-based hydrogels have seen practical application, but their viscoelastic behavior remains incompletely characterized because currently available precision techniques cannot fully resolve the underlying molecular architecture [20]. This is consistent with the multi-technique limitation identified in Section Advanced Analytical Techniques for Structure Elucidation: no single method provides complete structural resolution, and combining methods sufficiently to close this gap remains costly and technically demanding.

### 8.3. Complexity of Bioactivity Pathway

Seed mucilage has demonstrated antidiabetic, anti-inflammatory, and antioxidant activity (Section 6), but the underlying mechanisms of these effects remain incompletely characterized [40]. For example, cress seed mucilage has shown antidiabetic activity via yeast fermentation, but the precise mechanism of interaction between its antidiabetic oligosaccharides and host biological systems remains underexplored. This mechanistic gap is not merely descriptive; it is the reason the evidence-level caveats flagged (animal vs. human, in vitro vs. in vivo) matter: without mechanistic confirmation, health claims for seed mucilage bioactivities rest on associative rather than causal evidence.

### 8.4. Issues in Industrial Scale-Up

Translating laboratory-level mucilage research to industrial scale introduces substantial technical and financial challenges, since mucilage-based polymer design is both costly and highly quality-sensitive [219]. Quince seed mucilage combined with ZnO nanoparticles, for example, has been successfully developed into specialized burn-treatment bandages at laboratory scale, but scale-up requires resolving both technical and financial barriers before commercial deployment [220]. Similarly, *Ocimum basilicum* seed mucilage combined with montmorillonite for bio-nanocomposite food packaging films faces open questions of cost-effectiveness at industrial scale [221]. This scale-up challenge connects directly to the regulatory and commercialization barriers even where a laboratory-scale application is technically successful, the absence of standardized extraction/purification protocols and approved regulatory status compounds the difficulty of industrial translation.

### 8.5. Safety, Toxicology, and Quality Control

A substantive gap in the current seed mucilage literature, one that directly limits its translation into regulated food, pharmaceutical, and cosmetic products, is the near-absence of dedicated toxicological and safety-specific studies on seed mucilage itself, as distinct from the general food-safety literature on seeds broadly. Systematic evaluation of heavy-metal contamination (e.g., lead, cadmium, arsenic, mercury), pesticide residues, and microbial load specific to mucilage extracts, rather than to whole seeds or unrelated food matrices, is rarely reported alongside the yield, structural, or functional data reviewed in Section 3, Section 4, Section 5 and Section 6. Allergenicity is a related and more immediately actionable concern, since several of the seed sources discussed throughout this review are legumes with documented allergenic potential independent of their mucilage fraction [222,223]. Fenugreek (*Trigonella foenum-graecum*), prominently featured in antidiabetic activity and pharmaceutical excipient use, is a well-documented food allergen with clinically confirmed IgE-mediated cross-reactivity to peanut, including reported cases of anaphylaxis and occupational asthma following oral, inhalational, and dermal exposure to fenugreek seed powder [224,225]. This has direct practical consequences for the pharmaceutical and food applications proposed in Section 7: any formulation using fenugreek seed mucilage as an excipient or functional ingredient carries a genuine allergen-labeling obligation that is not addressed anywhere in the current manuscript, and this should be stated explicitly rather than left implicit.

Taken together, this absence of dedicated safety, purity, and quality-control data, compounded by the extraction-standardization gap (Section 8.1) and the regulatory approval barrier, represents a coherent, three-part translational bottleneck: without standardized extraction protocols, safety/purity data cannot be meaningfully compared across studies; without that comparability, regulatory bodies cannot efficiently evaluate GRAS/food-additive petitions; and without regulatory approval, industrial-scale investment in mucilage-based products remains commercially unattractive regardless of functional performance. Closing this loop, rather than any single technical advance, is likely the most consequential unmet need identified across Section 4, Section 7 and Section 8 of this review.

## 9. Future Perspectives

### 9.1. Structural Characterization Using Advanced Techniques

As established in Section 8.2, mucilage’s structural complexity remains incompletely resolved by current analytical methods. Future research is likely to focus on integrating spectroscopic, chromatographic, mass spectrometric, and advanced microscopy/imaging techniques (Section Advanced Analytical Techniques for Structure Elucidation) to achieve molecular-level resolution beyond what any single method currently provides. This level of understanding would directly enable structure-guided modification, for example, tuning mucilage molecular weight to engineer nanomaterials with enhanced emulsifying properties, improved colloidal stability, or optimized drug-release kinetics, extending the pharmaceutical applications outlined in Section 7.

### 9.2. Understanding Mucilage Bioactivities

Building on the mechanistic gaps identified in Section 8.3, future work should prioritize elucidating the specific molecular pathways underlying mucilage’s antioxidant, prebiotic, and immunomodulatory effects (Section 6), rather than continuing to document associative outcomes alone. Mucilage–gut microbiota interactions represent a particularly active area, given growing interest in prebiotic and probiotic product development. Beyond prebiotic applications, mucilage’s gel-forming and encapsulating properties (Section 5) position it as a promising delivery scaffold for bioactive peptides with antihypertensive, anticancer, or antioxidant activity, an application area not yet explored in the primary mucilage literature but well precedented for structurally similar polysaccharide hydrocolloids.

### 9.3. Sustainable Production Processes

Given seed mucilage’s expanding industrial role, developing genuinely sustainable, low-impact extraction processes remains a priority, directly addressing the extraction-standardization gap identified in Section 8.1. Microbial fermentation-based approaches offer one promising direction, as demonstrated by yeast fermentation used to generate antidiabetic oligosaccharides from cress seed mucilage, suggesting fermentation could be extended from bioactivity enhancement to extraction and modification more broadly. Biomass-conversion research from algal biopolymer sources offers a further parallel worth adapting to seed mucilage. Machine learning-guided process optimization represents an emerging and largely unexplored opportunity: given the many interacting extraction variables identified in Section 4 (temperature, solvent ratio, treatment time, power/frequency), data-driven optimization could substantially reduce the empirical trial-and-error currently required to identify optimal, species-specific extraction conditions.

### 9.4. Production of Diversified Biomaterials

**Figure 8:**
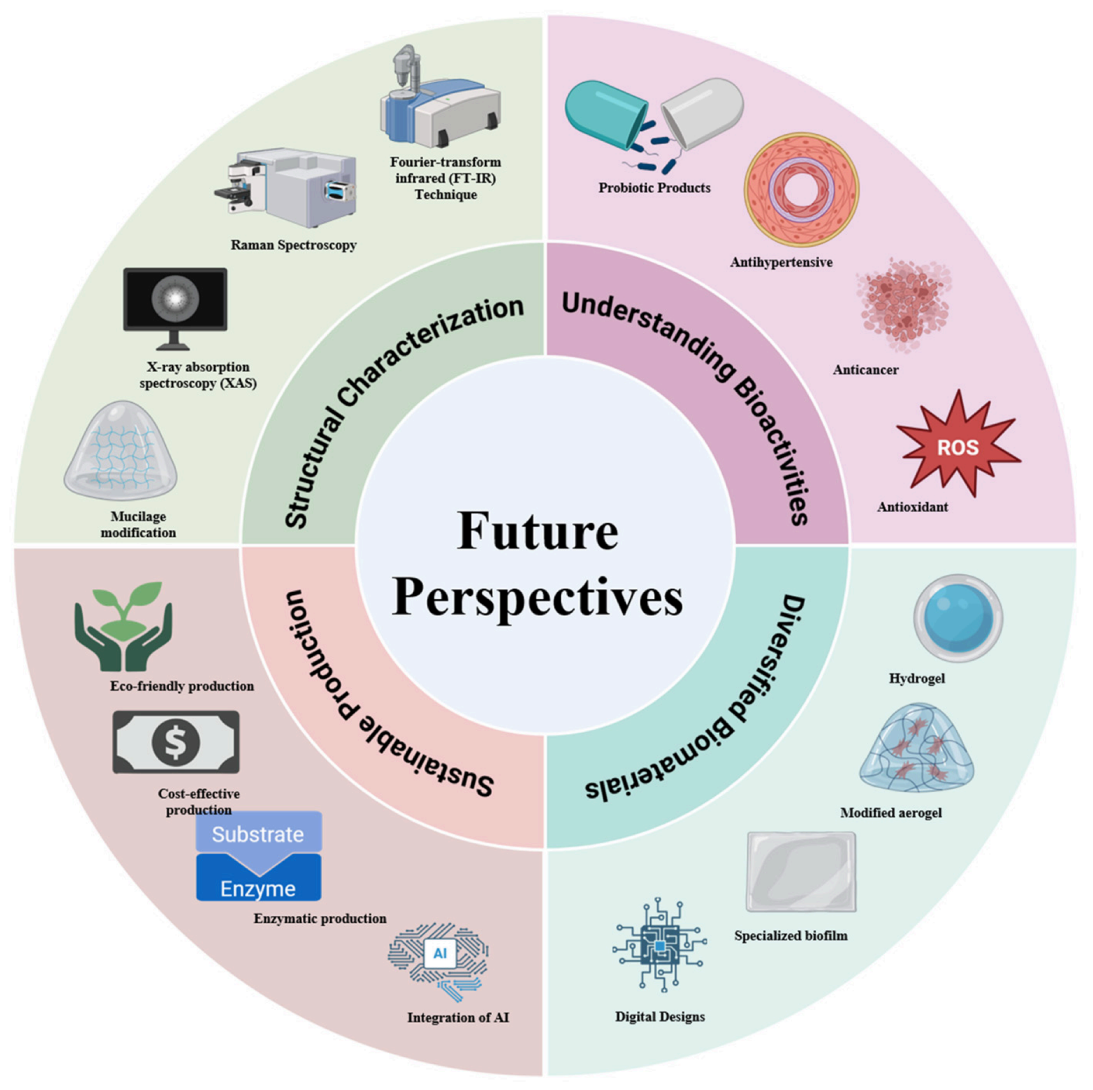
Future perspectives on seed mucilage from structural characterization to sustainable applications.

Mucilage’s gel-forming capacity, biodegradability, and bio-stability position it well for diversified biomaterial development, including aerogels, hydrogels, and biofilms with applications spanning the pharmaceutical and food sectors (Section 7). Exploring the viscoelastic behavior of mucilage-based hydrogels, for example, could yield improved wound-healing materials, building on the ZnO–mucilage bandage and montmorillonite–mucilage packaging composites already demonstrated at laboratory scale (Figure 8). Computer-aided materials design, including molecular simulation and machine learning-guided formulation, could accelerate this development pipeline considerably, though this remains a nascent application specific to mucilage biomaterials.

### 9.5. Commercialization, Regulatory Harmonization, and Clinical Validation

Beyond the technical advances above, the translational bottleneck identified across commercialization and clinical validation, the lack of standardized safety data, harmonized regulatory pathways, and approved food-additive status for most seed mucilages, represents a distinct and equally urgent research priority. Closing this gap will require coordinated effort on at least three fronts: (i) generating the mucilage-specific toxicological, allergenicity, and contamination data identified as largely absent in Section 8.5, structured to meet FDA and EFSA evidentiary requirements; (ii) conducting human clinical trials, rather than continuing to rely primarily on animal-model and in vitro evidence, to substantiate the nutraceutical and therapeutic claims increasingly made for mucilage-rich seeds; and (iii) developing techno-economic assessments of green extraction technologies (Section 4) at pilot and industrial scale, to establish the cost basis needed for genuine commercial investment. Without progress on these translational fronts, seed mucilage’s substantial technical promise, established throughout this review, is unlikely to convert into industrial adoption at meaningful scale.

## 10. Conclusions

Seed mucilage is a versatile, sustainable biopolymer with substantial and still largely unexploited industrial potential, representing a compelling intersection of evolutionary biology, green chemistry, and materials science. This review has synthesized the structural landscape of seed mucilage, largely characterized by pectins (RG-I, HG), hemicelluloses (xylans, glucomannans), and cellulose, and shown how molecular weight, uronic acid content, branching pattern, and microfibril organization directly govern its hydration, rheological, emulsifying, and foaming behavior (Section 3 and Section 5). Building on this structural foundation, we critically compared conventional and green extraction technologies, UAE, MAE, EAE, and SWE, establishing that no single technology dominates across all performance criteria, but that each offers a distinct trade-off between yield, molecular integrity, and processing cost (Section 4).

The functional and bioactive properties reviewed here, high water-holding capacity, tunable rheology, effective emulsifying and foaming behavior, and antioxidant, anti-inflammatory, prebiotic, and antimicrobial activity, support seed mucilage’s growing relevance across food, pharmaceutical, and cosmetic formulations (Section 5, Section 6 and Section 7). However, much of this bioactivity evidence currently rests on in vitro and animal-model studies rather than human clinical data, and the translation of these findings into approved nutraceutical or therapeutic products will require substantially stronger clinical validation before such claims can be considered established rather than promising. Similarly, seed mucilage’s industrial adoption remains constrained less by technical performance than by the absence of standardized extraction protocols, mucilage-specific safety and allergenicity data, and harmonized regulatory pathways, which are the barriers that are addressable, but currently underexplored relative to the structural and functional characterization that dominates the existing literature.

In addressing these dimensions together, molecular architecture, green process intensification, functional and bioactive performance, and the translational barriers that currently limit industrial deployment, this review aims to move beyond a descriptive catalog of seed mucilage sources toward the structure-informed, translationally grounded framework proposed in the Introduction. Realizing seed mucilage’s full potential as a sustainable alternative to synthetic hydrocolloids will depend on sustained, coordinated progress across structural characterization, extraction standardization, mechanistic and clinical validation, and regulatory harmonization, the five priorities outlined in Section 9.

## Figures and Tables

**Table 1:** Monosaccharide composition and structural polymers of selected seed mucilages.

Seed Name	Dominant Structural Compounds	Key Composition (% by Weight, Dry Basis)	References
Basil seed	Xylan	24.29	[51]
	Glucan	2.31	
	Glucomannan	43	
Chia seed	Glucose	19.6	[52]
	Galactose	6.1	
	Arabinose	9.6	
	Xylose	38.5	
	Galacturonic Acid	5.3	
	Glucuronic Acid	18.7	
Cress seed	Glucose	1	[53]
	Fructose	6.8	
	Arabinose	19.4	
	Rhamnose	1.9	
	Glucuronic Acid	6.7	
	Galactose	4.7	
Flax seed	Xylose	21.1–37.4	[54]
	Fructose	5–7.1	
	Galactose	20–28	
	Arabinose	9.2–13.5	
Balangu seed	Rhamnose	18.44	[55]
	Glucose	4.11	
	Galactose	33.54	
	Arabinose	37.88	
	Xylose	6.02	
Psyllium seed	Arabinose	23	[56]
	Xylose	75	
Qodume Shahri seed	Xylose	44.66	[57]
	Arabinose	31.99	
	Rhamnose	3.40	
	Galactose	12.77	
	Glucose	7.15	
Cassia seed	Mannose	77.2–78.9	[54]
	Glucose	6.3–7.1	
	Galactose	14.7–15.7	
Quince seed	Glucose	45.6	[58]
	Galactose	3.2	
	Xylose	10.7	
	Fructose	7.4	
Fenugreek seed	Galactomannan	22.57	[54]
	Galactose	0.25	
	Sucrose	0.79	
	Raffinose	0.50	
	Stachyose	2.84	

Values represent whole seed macronutrient composition. The 66% polysaccharide threshold for mucilage classification is applicable to purified/isolated mucilage fractions, not to whole seed composition.

**Table 2:** Common analytical techniques for the structural characterization of seed mucilages: principles, structural information, and comparative advantages.

Analytical Techniques	Principle	Structure Information	Advantages	Limitations	References
FTIR	Measures molecular vibrations through infrared absorption spectra	Identifies functional groups (–OH, –COOH, C–O–C, amide bands); confirms polysaccharide type	Rapid, non-destructive, minimal sample preparation	Overlapping peaks may limit resolution; semi-qualitative	[75,76,77,78]
NMR	Detects magnetic properties of atomic nuclei (^1^H, ^13^C) in magnetic field	Determine monomer type, linkage positions, anomeric configuration and ring form	Detailed molecular structure; non-destructive	Expensive equipment and complex data analysis	[82,84]
XRD	X-ray diffracted through crystalline structures	Identifies amorphous vs. crystalline regions; detects mineral phases	Reveals crystallinity and microstructure	Limited for amorphous samples	[75,88]
SEM	Uses electron beam to scan sample surface	Observes surface morphology, texture and moisture	High-resolution imaging; 3D morphology	Require vacuum and possible sample coating	[9,41,86]
TEM	Electron beam passes through ultra-thin section	Reveals nanostructure and fibril arrangement	High magnification and nanoscale image	Time-consuming and sample must be very thin	[87,89]
AFM	Scans surface using a sharp probe to measure atomic forces	Provides nanoscale topography and mechanical properties	Non-destructive and no coating needed	Slow imaging speed and limited scan area	[58]
HPSEC	Separate molecules by hydrodynamics volume	Determines molecular weight distribution and polydispersity	Accurate molecular size estimation	Requires calibration and pure solvents	[90,91]

Abbreviations: FTIR, Fourier transform infrared spectroscopy; NMR, Nuclear Magnetic Resonance; XRD, X-ray Diffraction; SEM, Scanning Electron Microscopy; TEM, Transmission Electron Microscopy; AFM, Atomic Force Microscopy; HPSEC, high-performance size-exclusion chromatography.

**Table 3:** Comparative overview of conventional and green extraction techniques for the extraction, purification, and drying of seed mucilages.

Technique Category	Method	Principle	Seed Type	Advantages	Disadvantages	References
**Conventional techniques**	HWE	Heating water (70–100 °C) to disrupt cell walls and enhance solubility of mucilage	Flaxseed, *Plantago major*	High yield, simple equipment, effective for most mucilages	High energy consumption, potential degradation of heat-sensitive compounds, long extraction times	[92,96]
	CWE	Hydrates seed in water at low temperatures (4–25 °C) to release mucilage via diffusion	Chia seeds	Preserves heat-sensitive bioactives, simple, low energy	Lower yield, longer extraction time compared to HWE	[98,130]
	AAE	Uses acidic or alkaline solutions to break down cell wall components and improve mucilage release	*Plantago major*, *Trigonella elliptica*	Can increase extraction yield, modifies functional properties	Requires strict pH control, potential chemical residue, can hydrolyse and degrade polysaccharides	[96]
**Green techniques**	UAE	Uses high-frequency sound waves to create cavitation bubbles, generating shear forces that break cell walls	Camelina seeds	Faster extraction, higher yield, lower temperature, energy-efficient	Potential for radical formation, equipment cost, optimization required for scale-up	[106,107]
	MAE	Uses microwave radiation to heat water molecules inside cells rapidly, causing internal pressure buildup and cell rupture	Basil, *Camelina sativa*	Very fast, high yield, improved functional properties, reduced solvent use	Non-uniform heating possible, capital cost, safety concerns	[113,114]
	EAE	Uses specific enzymes (e.g., cellulase, xylanase, pectinase) to selectively degrade cell wall polysaccharides	* Asplenium australasicum * (Bird’s nest fern)	Highly specific, mild conditions (pH, temperature), high purity, eco-friendly	High cost of enzymes, requires precise control of conditions, longer incubation times	[115,116]
	SWE	Uses water at high temperature (100–374 °C) and pressure to maintain liquid state, changing its polarity to act as a tunable solvent	Rambutan seeds (for polysaccharides)	Efficient for both polar/non-polar compounds, solvent-free (water only), fast	High equipment cost (pressure vessels), risk of degrading compounds at very high temperatures	[121,122,123]
** Purification **	Precipitation & Centrifugation	Mucilage is precipitated from the aqueous extract using solvents like ethanol or isopropanol, then separated via centrifugation	*Opuntia ficus-indica*, Most seed mucilages	Effective removal of impurities (proteins, minerals), concentrates mucilage	Use of large volumes of organic solvents, additional cost and safety concerns	[5,131,132,133]
**Drying**	Freeze drying	Sublimation of ice under vacuum to preserve porous structure	Chia, Flaxseed (for encapsulation), General use	Best for preservation of structure & bioactivity.	High energy cost	[97,134]
	Spray drying	Atomization into hot air	Chia, Flaxseed	Fast and scalable	High temp can degrade product	[131,135]
	Oven drying	Conventional heating	Chia, Flaxseed	Low cost and simple	Significant degradation of quality	[132,135]
	Vacuum drying	Moisture removal under reduced pressure	Most seeds mucilage	Low cost and simple	Slow process	[136]
	Air drying	Moisture removal by natural air	Most seeds mucilage	Simple and easy	Time-consuming, low yield	[136]

Abbreviations: HWE, hot-water extraction; CWE, cold-water extraction; AAE, Acid/Alkaline Extraction; UAE, ultrasound-assisted extraction; MAE, microwave-assisted extraction; EAE, enzyme-assisted extraction; SWE, subcritical water extraction.

**Table 4:** Bioactive properties and health implications of seed mucilage.

Bioactive Property	Mechanism of Action	Seed Sources	Observed Effects	References
Antioxidant activity	Scavenging free radical (DPPH, ABTS Assays), reducing power, metal chelation	*Linum usitatissimum* (Flax), *Plantago ovata* (Psyllium), *Ocimum basilicum* (Basil), *Salvia hispanica* (Chia), *Ximenia americana*	Strong radical scavenging activity due to presence of phenolics, polysaccharide hydroxyl groups and uronic acid	[165,172,173]
Ant-inflammatory activity	Inhibition of pro-inflammatory mediators (COX, LOX enzymes, TNF-α, IL-6)	*Hibiscus esculentus* (Okra), *Lepidium sativum* (Garden cress)	Reduced production of inflammatory cytokines; potential role in wound healing and gut health	[17,174,175]
Antimicrobial activity	Disruption of microbial cell wall/membrane; interference with metabolism	*Plantago major*, cress seed (*Lepidium sativum* L.)	Effective against Gram-positive and Gram-negative bacteria; synergistic effect with phenolic compounds	[17,165,176]
Antidiabetic activity	Attenuates glucose diffusion and absorption; enhances insulin sensitivity	*Plantago ovata* (Psyllium), *Salvia hispanica* (Chia), *Trigonella foenum-graecum* (Fenugreek)	Mucilage reduces postprandial glucose levels; improves glycemic control in animal model	[165,177]
Anticancer activity	Induction of apoptosis; inhibition of oxidative stress and tumor growth	*Flax*, *Fenugreek*, *Chia*	Demonstrated cytotoxicity against certain cancer cell lines through ROS modulation	[171,174]
Prebiotic and gut health	Promotion of gut microbiota; enhancement of SCFA production	*Psyllium*, *Chia*, *Basil*	Increases growth of *Lactobacillus* and Bifidobacterium; improves gut barrier function	[178,179]
Cholesterol-lowering activity	Bile acid binding and inhibition of lipid absorption	*Psyllium*, *Flax*, *Fenugreek*	Reduce total and LDL cholesterol in animal and human studies	[1,3]
Wound healing and skin protection	Moisture retention, anti-inflammatory and antioxidant actions	*Okra*, *Flax*, *Basil*	Enhances epithelial regeneration and protects against oxidative skin damage	[13,180,181]

Abbreviations: DPPH, 2,2-Diphenyl-1-picrylhydrazyl; ABTS, 2,2′-Azino-bis (3-ethylbenzothiazoline-6-sulfonic acid); COX, Cyclooxygenase; LOX, Lipoxygenase; TNF-α, Tumor Necrosis Factor-alpha; IL-6, Interleukin-6; LDL, Low-Density Lipoprotein; ROS, Reactive Oxygen Species; SCFA, short-chain fatty acid.

## Data Availability

No new data was created or analyzed in this study. Data sharing is not applicable to this article.

## Abbreviations

The following abbreviations are used in this manuscript:

AAE	Acid/Alkali-Assisted Extraction
AFM	Atomic Force Microscopy
CWE	Cold Water Extraction
EAE	Enzyme-Assisted Extraction
FTIR	Fourier-Transform Infrared Spectroscopy
HG	Homogalacturonan
HPSEC	High-Performance Size-Exclusion Chromatography
HWE	Hot Water Extraction
MAE	Microwave-Assisted Extraction
NMR	Nuclear Magnetic Resonance
RG-I	Rhamnogalacturonan I
SCFA	Short-Chain Fatty Acids
SEM	Scanning Electron Microscopy
SWE	Subcritical Water Extraction
TEM	Transmission Electron Microscopy
UAE	Ultrasound-Assisted Extraction
XRD	X-ray Diffraction
ZnO	Zinc Oxide
